# Anterior Cutaneous Nerve Entrapment Syndrome: Two Case Reports of the Forgotten Diagnosis After Bariatric Surgery

**DOI:** 10.7759/cureus.8499

**Published:** 2020-06-08

**Authors:** Hendrika Smelt, Sjaak Pouwels, J.A. Apers, Mohammed Said, Johannes Smulders

**Affiliations:** 1 Surgery, Catharina Hospital, Eindhoven, NLD; 2 Intensive Care Medicine, Elisabeth-Tweesteden Hospital, Tilburg, NLD; 3 Surgery, Franciscus Gasthuis & Vlietland, Rotterdam, NLD

**Keywords:** anterior cutaneous nerve entrapment syndrome, abdominal wall pain, bariatric surgery, neurectomy, diagnostic injection

## Abstract

Unexplained abdominal pain is an increasing phenomenon after laparoscopic bariatric surgery, with an occurrence of 7.4%. The pain could be explained by the anterior cutaneous nerve entrapment syndrome (ACNES). However, the incidence of ACNES after laparoscopic bariatric surgery is unclear. We report the cases of two patients with unexplained abdominal pain after laparoscopic bariatric surgery and a significant delay in the diagnosis of ACNES. In both cases, clinical signs of ACNES were demonstrated by a centralized trigger point in the abdominal wall and specific neuropathic aspects during examination. Both patients were temporary pain-free after a diagnostic local lidocaine injection. A neurectomy was performed in both cases, after which they remained pain-free. There was a significant delay (six months and three years, respectively) in the diagnosis of ACNES, and many additional imaging procedures including a diagnostic laparoscopy were performed. ACNES is difficult to diagnose due to its relatively unknown entity.

This case report confirms that the diagnosis of ACNES is still frequently overlooked as a cause of chronic abdominal pain. Earlier diagnosis recognition can probably prevent unnecessary investigations and may improve the quality of life in bariatric patients with unexplained abdominal pain.

## Introduction

Unexplained abdominal pain is an increasing phenomenon after laparoscopic bariatric surgery, with an estimated occurrence of 7.4%. Chronic abdominal pain is associated with a reduced health-related quality of life (QoL). Chronic abdominal pain could be explained by the anterior cutaneous nerve entrapment syndrome (ACNES) [1,2]. However, the exact incidence of ACNES after laparoscopic bariatric surgery is unclear. The diagnosis is still frequently overlooked, and patients are subjected to prolonged investigation [3-7]. Here we report the cases of two patients with unexplained abdominal pain after laparoscopic bariatric surgery and a delay in the diagnosis of ACNES. In these cases, the diagnosis of ACNES was made after a long clinical pathway and the absence of abnormalities in blood analysis and radiological imaging.

## Case presentation

Case 1

A 36-year-old woman underwent a laparoscopic sleeve gastrectomy (LSG) in 2017. She underwent a laparoscopic revision to a Roux-en-Y gastric bypass (RYGB) in August 2019 due to persistent gastroesophageal reflux disease (GERD) with reflux esophagitis. Her surgical history included a laparoscopic cholecystectomy in 2018. A multivitamin supplement was prescribed, and no other medication was used.

She presented six weeks after surgery with severe colicky pain diffused in the right upper abdomen for several times. Her food intake was moderate due to nausea, vomiting, flatulence, and regurgitation of food despite the use of proton pump inhibitor. Physical examination showed normal hemodynamic values (heart rate of 75 beats per minute, temperature of 36.9ºC, blood pressure of 135/74 mmHg, oxygen saturation of 98%, respiratory rate of 14 breaths per minute) and pressure pain in the right upper abdomen. An esophagogastroduodenoscopy and ultrasound of the upper abdomen were performed, which showed no abnormalities. Additional dietary treatment was started due to analysis of dumping syndrome and other food intolerances. Two weeks later (eight weeks postoperatively), she came back with persistent unsustainable pain (more than four hours) in the right upper abdomen. There was no flatulence, nausea, or regurgitation since dietary adjustments were initiated. Laboratory results showed increased liver enzymes (Table 1). An abdominal computed tomography (CT) was performed, which showed no abnormalities. Afterward, a magnetic resonance cholangiopancreatography was performed due to increased liver enzymes, which showed no abnormalities. Ten weeks postoperatively, she came back, and all laboratory investigations showed values within normal range.

**Table 1:** laboratory results case presentation 1 CRP, C-reactive protein; ASAT, aspartate aminotransferase; ALAT, alanine aminotransferase; AF, alkaline phosphatase; GGT, gamma glutamyl transferase; GFR, glomerular filtration rate

	Reference value	8 weeks postoperatively	10 weeks postoperatively
Hemoglobin	7.5-10.0 mmol/L	8.8	
Hematocrit	0.35-0.45 L/L	0.43	
Leukocytes	4.0-10.0 x 10^9^/L	7.7	
CRP	<6.0 mg/L	< 6.0	
Bilirubin	<17 µmol/L	15	8.1
ASAT	<30 U/L	80	24
ALAT	<40 U/L	290	38
AF	40-120 U/L	354	110
GGT	<40 U/L	66	31
Kreatinin	50-100 µmol/L	58	
GFR	>90 mL/minute	>90	
Lipase	<60 U/L	20	

The abdominal complaints were less severe and no more acute pain moments had occurred for three weeks. Her complaints were initially interpreted as a passed gallstone in the common bile duct. However, there was an unexplained specific aspect of pain; she could pinpoint the pain with one finger directly lateral from the laparoscopic scar in the right upper abdomen. It seems to be position-dependent. Repeated physical examination showed a clear trigger point with local sensory disturbance over 3 cm around the trigger point (hyperesthesia and allodynia). The pinch test and Carnett’s sign were positive. ACNES was suspected, and a diagnostic cutaneous 1% lidocaine injection (5 mL) was given. Afterward, she was completely pain-free for a day and a half. The lidocaine injection was repeated after two weeks. She was completely pain-free for a few days again. A surgical neurectomy was performed, after which she was pain-free. She was still pain-free three months later.

Case 2

A 28-year-old woman underwent a LSG in December 2014 and a laparoscopic revision to a RYGB in April 2015 because of passage problems due to a stenosis of the sleeve. She was a smoker and had no medical history. A multivitamin supplement was prescribed, and no other medication was used.

After RYGB, she recovered well. She canceled several control appointments for a period of 2.5 years without a clear explanation. Three years after RYGB, she presented with monthly colicky pain attacks in the right upper abdomen for five months (pain score: 9-10). The pain remained for one to two hours, and afterward she was pain-free. The pain became progressive. Besides that, she had postprandial retrosternal pain, which is accompanied by dyspepsia (reflux, regurgitation of food, and nausea). An ultrasound abdomen was already performed at the request of the general practitioner, which showed no abnormalities; no gallstones were present. Physical examination showed normal hemodynamic values (blood pressure of 125/85 mmHg, heart rate of 68 beats per minute, respiratory rate of 12 breaths per minute, oxygen saturation of 99%, temperature of 37.1ºC) and pressure pain in the right upper abdomen and epigastric region. Laboratory results showed no abnormalities. An abdominal ultrasound was repeated and showed no abnormalities again. Helicobacter pylori serology was negative. An esophagogastroduodenoscopy was performed, which showed an abnormal mucosa of the distal esophagus, and the pathology of a biopsy showed Barrett’s metaplasia. Triple therapy with ranitidine, pantoprazole, and sucralfate was started, and the patient was registered for the clinical Barrett follow-up program. Initially, the symptoms initially seemed to decrease. However, one month later, she came back with progressive pain. Physical examination shows tachycardia (hart rate of 104 beats per minute), a temperature of 38.2ºC, and pressure pain diffused in the upper abdomen. No abnormalities were found in the laboratory results again. A CT of the abdomen was performed subsequently and showed no signs of internal herniation and no abnormalities. A diagnostics laparoscopy was performed because of the unsustainable pain, but no internal herniation was found, mesenteric space was still closed, and Peterson’s space was closed. Other perioperative findings were normal: anatomy and position gastroenterostomy and enteroenterotomy were normal and no abnormalities in the entire intestines were seen. Afterward, the pain attacks decreased to one to two times in one hour and were posture-dependent. Typically, the pain seems to centralize more to one point at the right upper abdomen (Th8), and typically she reported that she could not wear tight clothes on the skin. Physical examination showed a clear punctum maximum, and the pinch test and Carnett’s sign were positive. The diagnosis of ACNES was made, and she received a lidocaine 1% injection (5 mL) at the trigger point beneath the fascia. The pain has decreased significantly for two days after injection. Afterward, the pain was acceptable, and no unsustainable pain attacks occurred. She received a second lidocaine injection one week later, after which she was pain-free for two days. Subsequently, a neurectomy was performed and she was pain-free permanently afterward. One year later, she was still pain-free. Her QoL has improved enormously, and she was able to lead a normal life again without any limitations.

She has had her follow-up control moment at the gastrointestinal-liver department: pantoprazole was continued, ranitidine and sucralfate were both stopped. The next control moment with esophagogastroduodenoscopy was planned at the three-year follow-up.

## Discussion

These case reports confirm that ACNES is still an infrequently overlooked diagnosis and that most patients are subjected to prolonged investigation during the analysis of chronic abdominal pain. In the first case, logically, the focus was initially on intra-abdominal causes, because intra-abdominal complications are common after bariatric surgery. In the second case, the diagnosis Barrett’s esophagus (BE) was found, for which triple eradication therapy was started. A discrepancy between her typical complaints and the findings of the esophagogastroduodenoscopy was seen. The preoperative tachycardia in case 2 can be indicated by severe pain. The elevated temperature can be discussed because this symptom is not typical for ACNES or a BE. However, no cause for this elevated temperature was found on radiological examinations and during the laparoscopy. In addition, a possible double diagnosis was not considered earlier because her complaints decreased considerably when the triple therapy was started. On the other hand, BE is not always symptomatic nor is severe pain a common symptom [8]. ACNES symptoms were examined after the patient returned with unbearable pain and after she underwent a diagnostic laparoscopy without any abnormalities.

Both patients of this case study initially suffered from nausea, bloating, and flatulence. Therefore, these complaints were primarily considered an intra-abdominal cause. However, physicians should be aware that pseudo-visceral symptoms such as nausea, bloating, reduced appetite, and altered defecation may also be related to ACNES [9]. The presence of these symptoms does not exclude the diagnosis of ACNES [9]. There was a typical delay in the diagnosis of ACNES in both cases (six months and three years for cases 1 and 2, respectively) This is a common aspect of ACNES, as described by Boelens et al., who reported a median delay to diagnosis of 13 months [3]. However, this suggests that there is no direct correlation between the bariatric surgery (incisions scars adhesions) and ACNES. The entrapped nerves causing the pain cannot be visualized using imaging or functional techniques. Therefore, ACNES is rightfully termed a clinical diagnosis [6]. Difficulty in diagnosing ACNES leads to excessive and unnecessary blood tests and imaging, and multiple hospital visits. Associated costs, and socio-economic and psychosocial consequences of ACNES are considerable [6,10].

Anatomy

**Figure 1:**
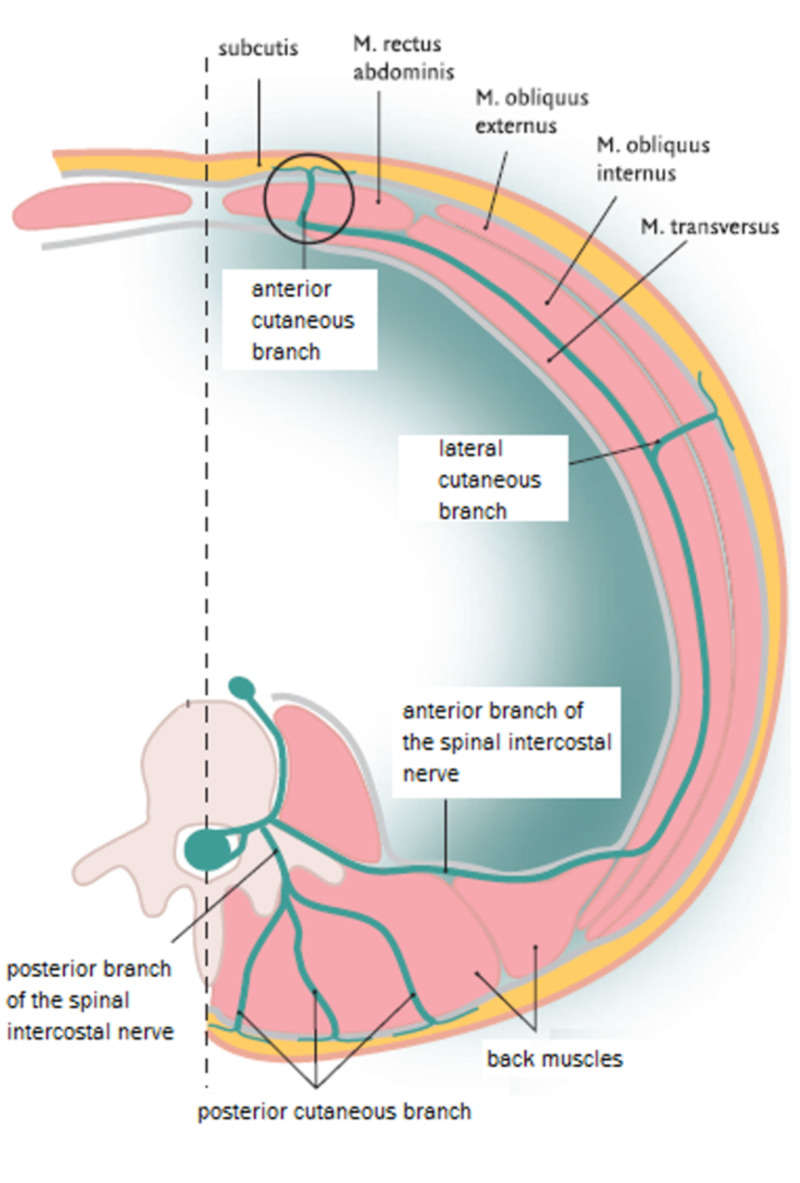
Course and distribution of an intercostal nerve at the abdominal level

**Figure 2:**
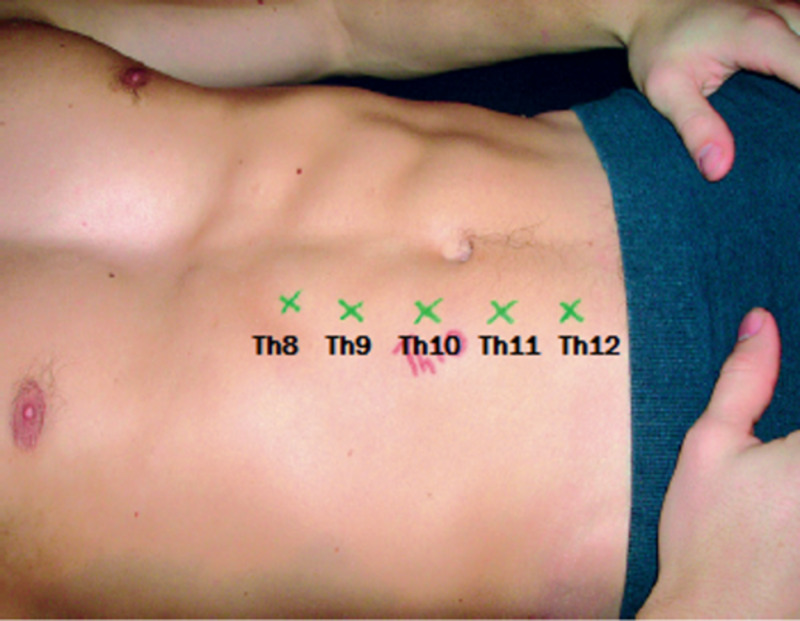
Five clinically identifiable points were the anterior intercostal nerve branches (Th8-Th12) penetrating the rectus abdominis muscle

TThe abdominal wall skin receives its sensory innervation from the intercostal nerves (Th8-Th12), and these nerves run between the internal oblique and transversus abdominis muscle, up to the point where they reach the rectus abdominis. The anterior cutaneous intercostal nerve branches penetrates the back of the rectus abdominis muscle at a corner of almost 90 degrees (Figure 1), mostly located at five foramina points (Figure 2), on both the left and right lateral borders of the rectus abdominis muscle [5,7]. Afterward, these nerves pass through the subcutaneous fascia ending in the terminal skin branches [5,6].

 

Diagnostics in patients with ACNES

The maximum pain point is mostly situated between the midline and the lateral edge of the rectus abdominis muscle on the left or right side. Usually, the maximum pain point can be located with one finger. Some patients had an area of several centimeters around this trigger point with sensory disturbance such as hypoesthesia, allodynia, or hyperalgia, which is highly suggestive for ACNES [5,11]. Pain increases due to muscle tensing, movement, or exercise, or is related to posture [6]. A positive pinch test and positive Carnett’s sign are typically suggestive for the diagnosis of ACNES during physical examination [5,6]. In the presence of these symptoms and the absence of abnormalities in blood analysis and additional research, a patient has a diagnosis of ACNES until proven otherwise [3]. The diagnosis can be confirmed by a local anesthetic injection at the trigger point [11]. Even short during effect confirms local entrapment in the abdominal wall. Thirty-three percent of the patients remained permanently pain-free after the injection. Seventy-six percent of the patients reported to be satisfied 18 months after the injection [11]. So far, no studies are available on the treatment of ACNES in bariatric patients. This mechanism must be further investigated in the bariatric patient population.

A successful neurectomy was performed in both cases, and both patients were pain-free afterward. Seventy-one percent of the patients reported to be very satisfied six weeks after a neurectomy in the study by Boelens et al. [11]. The study of Van Assen et al. showed a short-term success rate of 70% and a long-term success rate of 61% after a neurectomy [12]. A neurectomy should therefore be performed if the therapeutic injection has an insufficient effect. However, approximately 30-40% of the patients have persistent complaints of pain after the neurectomy [12]. Therefore, the diagnosis of (persistent) ACNES should not necessarily be reconsidered if symptoms persist.

Possible causes of ACNES before and after bariatric surgery

Specific conditions in relation to an increased abdominal compliance (previous pregnancy, laparoscopy, and gynoid fat distribution) and a decreased abdominal compliance (obesity, android fat distribution) were described by Malbrain et al. [13]. A decreased abdominal wall compliance due to (morbid) obesity caused by increased intra-abdominal pressure will push the abdominal wall anteriorly and raise pain from an entrapped nerve. Besides that, the intra-abdominal and subcutaneous fat pads also expand the abdominal wall [14,15]. The most common condition associated with abdominal wall pain was obesity, and a body mass index above 30 kg/m^2^ was seen in 41% of the study patients [14].

Bariatric surgery results in significant weight reduction, improvement in obesity-related comorbidities, and significant changes in abdominal composition and fat distribution. Abdominal fat is divided into intra-abdominal and subcutaneous fat [15]. Weiss et al. described a decrease of 32% of subcutaneous fat and a decrease of 35% in visceral fat six months after bariatric surgery [15]. The strongest relation between absolute and relative changes in visceral and subcutaneous fat was demonstrated for the excess weight loss (EWL) after bariatric surgery. EWL was associated with a reduction in subcutaneous and intra-abdominal fat depots, more strongly than other anthropometric measures. However, the influence of changes in body composition and fat distribution on the development of ACNES is still unclear.

Our hypothesis is that the intra-abdominal pressures will decrease and the abdominal wall compliance will increase after bariatric surgery. Possibly, weak spots might arise in the anterior abdominal wall muscles due to fast weight loss [4]. Besides that, many patients move and exercise more and become more active in daily life after bariatric surgery. As a result, muscles are rebuilt and strengthened, which might cause another intra-abdominal pressure on the abdominal wall because the abdominal wall will push posteriorly and raise pain from an entrapped nerve. This mechanism can affect the development of ACNES.

The current literature even suggests that gastrointestinal symptoms can cause abdominal wall pain. In patients with bloating or distention of intestines, which is common after bariatric surgery, the response to increased intra-abdominal volume is exaggerated and is associated with muscular dystonia of the abdominal wall and might cause nerve entrapment subsequently [14,16]. This is another theory of a potential cause of this specific condition. Forceful contractions and intermittent abdominal wall distension during bloating can cause exert strain on the abdominal wall structures. The muscles of the anterior abdominal wall are relaxed, but the abdominal wall fasciae are stretched in patients with complaints such as bloating [14]. Gastrointestinal complaints or intestinal distention are common after bariatric surgery, especially in patients with dumping or fructose/lactose-intolerance.

This case report emphasizes the importance of thorough physical examination in patients with unexplained abdominal pain. ACNES should always be considered in the differential diagnosis of chronic abdominal pain in the absence of other potential causes [17]. Therefore, It is also important to differentiate between visceral pain by dysfunctional organs and parietal pain with specific clinical abnormalities in the abdominal wall [5,11]. Analyses of ACNES characteristics must be added to the physical examination standardly, which may be contribute to a faster diagnosis. An accelerated diagnostic pathway in patients with suspected ACNES, searching for a trigger point in the rectus abdomens muscle with a simple diagnostic lidocaine injection, might cut costs by reducing the number of unnecessary investigations [3,11].

## Conclusions

We described two cases of ACNES after laparoscopic bariatric surgery. ACNES was initially not included in the differential diagnosis. Intra-abdominal causes were initially suspected due to a combination of several complaints in both cases. Therefore, the pinch test and Carnett’s sign were not performed during the primary physical examination, which caused delay in the diagnosis of ACNES. These case reports confirm that ACNES is still frequently overlooked and that patients are subjected to prolonged investigation. If ACNES is suspected, based on clinical signs and symptoms, a cutaneous lidocaine injection should be administered. Earlier recognition of ACNES can probably prevent prolonged and unnecessary investigation and may improve the QoL in bariatric patients with unexplained abdominal pain.
